# The Comparative Field Evaluation of Four Different Traps for Mosquito Surveillance in the Republic of Korea

**DOI:** 10.3390/insects15070531

**Published:** 2024-07-13

**Authors:** Hak Seon Lee, Byung-Eon Noh, Seong Yoon Kim, Hyunwoo Kim, Hee Il Lee

**Affiliations:** Division of Vectors and Parasitic Diseases, Korea Disease Control and Prevention Agency, 187 Osongsaengmyeong 2-ro, Osong-eup, Heungdeok-gu, Cheongju 28159, Republic of Korea; hslee8510@korea.kr (H.S.L.); nbudia@korea.kr (B.-E.N.); gunbo0402@korea.kr (S.Y.K.); hyunwookim@korea.kr (H.K.)

**Keywords:** mosquito traps, efficiency, comparison, Republic of Korea

## Abstract

**Simple Summary:**

Mosquito collection traps are an essential tool for monitoring mosquito density and species distribution. Various traps are used in mosquito surveillance projects in the Republic of Korea. However, there is a lack of comparative analysis of these traps. Therefore, we evaluated four traps that are widely used for mosquito surveillance. BLT showed superior collection efficiency in terms of the number of collected individuals and species evenness, whereas BGT showed the highest species diversity among all the traps. DMS is well adapted to urban areas and has an advanced function that can automatically count the number of mosquitoes. In our summary, we provide information about the traps and guidance for planning new mosquito surveillance projects.

**Abstract:**

Monitoring mosquito populations is essential for controlling mosquito-borne diseases, and the selection of mosquito traps should be tailored to specific surveillance objectives. Here, we tested four mosquito traps for their efficiency and applicability: the Nozawa-style black light trap (BLT), BG-sentinel trap II (BGT), UV-LED Blackhole Plus Mosquito Buster trap (LED), and digital mosquito monitoring system (DMS). The traps were rotated weekly for a 24 h cycle at the same location for 13 weeks. Overall, 1649 female mosquitoes belonging to seven genera and sixteen species were collected by the traps. The traps exhibited differences in both the number of collected individuals and species composition. The BLT showed superior collection efficiency in terms of the number of collected individuals and species evenness, whereas the BGT showed the highest species diversity among all the traps. Thus, the BLT and BGT are the best choices for effective mosquito surveillance based on trap performance. Additionally, despite the relatively low efficiency of the LED and DMS observed in this study, the LED is known to be efficient when used for indoor conditions such as cowsheds, while the DMS has an advanced function that can automatically count the number of mosquitoes. Thus, our findings provide significant guidelines for planning new mosquito surveillance projects in the ROK.

## 1. Introduction

The systematic monitoring of the seasonality and abundance of mosquito populations is crucial for integrated vector management [1]. Mosquito collection traps are an essential tool for monitoring mosquito density and species distribution within an area. The New Jersey light trap, developed in 1932, was the old standard for mosquito surveillance [2]. Since then, several traps have also been invented and tested. Currently, the most representative and commonly used trap is the Centers for Disease Control and Prevention (CDC) light trap, developed in the 1960s [3]. Additionally, attractants such as carbon dioxide (CO_2_) have been used to enhance the effectiveness of mosquito traps [4]. In the late 1990s, new concepts for trapping were developed, such as the counterflow technique and colored light-emitting diodes. The counterflow geometry (CFG) trap is designed to catch mosquitoes that avoid air streams through vigorous flights [5]. The Mosquito Magnet is a well-known trap that uses a similar counterflow technology. Colored light-emitting diodes have shown potential as alternative light sources compared to ultraviolet lamps [6]. In the middle of the 2000s, the BG-Sentinel trap (BGT) became a popular tool for monitoring *Aedes* spp. such as *Ae. aegypti* and *Ae. albopictus*, which are primary vectors of mosquito-borne diseases [7,8]. Since the 2010s, traps that count the number of collected mosquitoes and transmit data remotely, such as the recently developed digital mosquito monitoring system (DMS) and BG counter [9,10], have received significant attention.

The Republic of Korea (ROK) lies in the temperate zone and has been reported to have fifty-six species of mosquitoes from nine genera [11]. Japanese encephalitis, transmitted by *Culex tritaeniorhynchus*, is considered an indigenous disease in the ROK. Although the number of patients has significantly decreased due to the government’s vaccine policy that began in 1985, 20 cases on average were reported annually from 2013 to 2022 [12,13]. Vivax malaria, transmitted by *Anopheles*, was widespread in the nation until the 1950s [14]. The World Health Organization (WHO) declared the ROK malaria-free in 1979, as a result of the government’s eradication project [15]; however, since its reemergence in 1993, approximately 460 cases have been reported annually from 2013 to 2022 [13,16]. Moreover, *Ae. albopictus*, which is a vector of flaviviral diseases, such as dengue fever, yellow fever, Zika virus infection, and *Cx. pipiens*, which transmit West Nile fever, are distributed throughout the country [17].

Owing to these disease outbreaks, the Korea Disease Control and Prevention Agency (KDCA) continues to conduct mosquito surveillance projects. To monitor Japanese encephalitis vector mosquitoes, the Nozawa-style black light trap (referred to as BLT hereafter) has been installed in 11 cowsheds across the country since 1975 [18]. In addition, the surveillance of the vivax malaria vector, which commenced in 2009, has been monitored using the UV-LED Blackhole Plus Mosquito Buster trap (LED hereafter) across 50 private homes and military base sites located in vivax malaria-endemic areas [19]. Furthermore, climate change and variability have been reported to influence vector-borne disease epidemiology [20]. For instance, in the ROK, Lee et al. [21] predicted that dengue fever may spread to the southern part of the country owing to the effects of globalization and climate change. To cope with this situation, the KDCA has established surveillance systems comprising 16 local centers to monitor mosquito population density, including vector species and related pathogens. The centers cover the entire country, with 36 mosquito collection sites in 30 regions using BLTs and BGTs [17]. Additionally, the DMS was introduced in 2019 to improve the efficiency of the surveillance system [22].

Studies on mosquito traps have been conducted in several regions of the ROK, with a primary focus on medically important vector species. Burkett et al. [23] conducted a comparative analysis of six commercially available mosquito traps and observed that the Mosquito Magnet was the most effective among them at capturing *Anopheles sinensis* sensu lato in malaria-endemic areas. In a similar study by Kim et al. [24], the light-emitting diode trap showed better efficiency than the BLT for trapping the *Anopheles hyrcanus* group in cowsheds in malaria-endemic areas. Similar comparative trap studies have been conducted in Asia, Europe, North America, South America, and Oceania, focusing on invasive or vector-competent species [25,26,27,28,29,30,31,32,33]. These studies present comparative data on the efficacy of traps under different field conditions and target species. However, a comparative field evaluation of newly developed devices for counting captured mosquitoes is missing. Although various traps are used in mosquito surveillance projects in the ROK, there is a lack of comparative analysis of these traps, including the DMS.

Therefore, in this study, we compared four traps, the BLT, BGT, LED, and DMS, for their efficiency in collecting mosquitoes. This study aims to provide useful information for selecting appropriate devices in mosquito research and long-term surveillance programs.

## 2. Materials and Methods

### 2.1. Study Site

**Figure 1:**
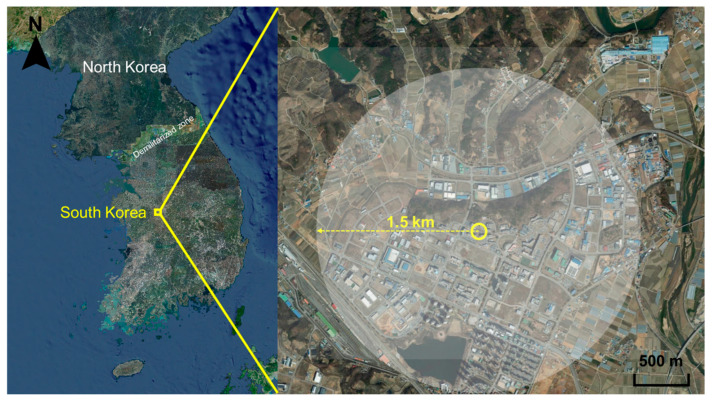
Map of the study site where traps were installed. The left map presents a broad view of the site’s location, while the right map provides a detailed view (image sourced from the Korea Statistical Information Service).

The study was conducted at Osong Biovalley (127°19′35.42″ N, 36°38′24.05″ E), located in Osong-eup, Heungdeok-gu, Cheongju-si, ROK. Diverse environments, including apartment complexes, industrial complexes, farming areas, artificial ponds, detention ponds, reservoirs, and hills, were present within a 1.5 km radius of the study area (Figure 1). The annual average temperature in the study region is 12.4 °C, and annual rainfall is 1220.9 mm.

### 2.2. Traps and Collection

**Figure 2:**
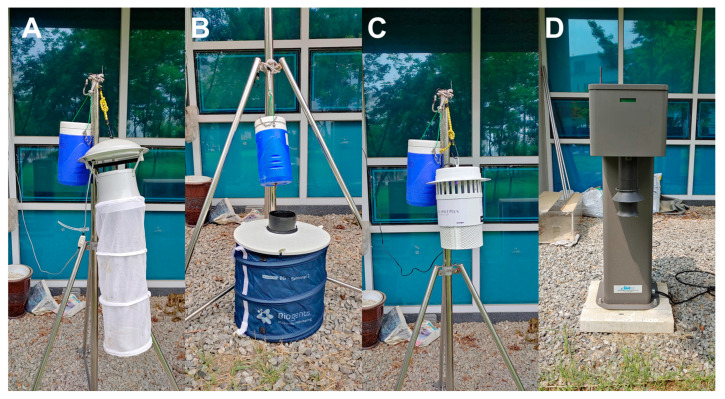
Traps and settings tested for efficiency comparison. (**A**) black light trap; (**B**) BG-sentinel trap II; (**C**) light-emitting diode trap; (**D**) digital mosquito monitoring system.

The following is a description of each trap and its location relative to the ground. The BLT (Shin-young Comm. System, Namyangju, Republic of Korea), which is similar to the CDC black light trap and the New Jersey light trap, was placed at a height of 1.5 m from the ground. This trap attracts mosquitoes using two fluorescent ultraviolet lamps (Figure 2A). The BGT with a BG-lure chemical bait (Biogents AG, Regensburg, Germany) was placed on the ground (Figure 2B). The LED (Biotrap, Gunpo, Republic of Korea), which uses two UV light-emitting diode lamps to attract mosquitoes, was placed in the same manner as the BLT (Figure 2C). A dispenser containing approximately 1.5 kg of dry ice was equipped with the three traps as additional bait. Finally, the DMS (E-TND Co. Ltd., Gwangju, Republic of Korea) is a ground-mounted trap with a counterflow intake and a CO_2_ outlet at a height of 1 m above the ground (Figure 2D). CO_2_ was emitted at approximately 300 mL/min from 7:00 pm to 3:00 am from a cylinder, controlled by two regulators and a computer. For the DMS trap, the number of mosquitoes collected can be checked in real time using a laser sensor. The traps were placed in the same spot. Each mosquito trap was rotated sequentially in a 24 h cycle and operated at least once within a week. Mosquito collection was conducted for a total of 13 weeks from 31 May to 27 August 2021.

### 2.3. Mosquito Identification

The collected mosquitoes were then transported to the laboratory. Female mosquitoes were identified under an Olympus SZ-61 stereomicroscope (EVIDENT Corp., Tokyo, Japan) using taxonomic keys at the species level [34]. However, anopheline mosquitoes were identified to the genus level due to the difficulty in species identification based on morphological characteristics.

### 2.4. Data Analysis

A non-parametric Kruskal–Wallis test was used to confirm the difference in the efficiency of each trap. A *p* value < 0.05 was considered statistically significant. In case of a statistically significant difference, trap-versus-trap comparisons were performed using the Mann–Whitney U test, and the significance level was determined using the Bonferroni method. Analyses were performed using PASW Statistics for Windows version 18.0 (IBM SPSS Inc., Armonk, NY, USA).

A biodiversity analysis was performed using Simpson’s diversity index (1-D) and evenness (E) to evaluate the species richness and abundance in each trap [35,36].

## 3. Results

In total, 1649 female mosquitoes belonging to seven genera and at least sixteen species, including *Anopheles* spp., were captured by the four traps. The highest number of female mosquitoes, 511 individuals belonging to 15 species, was collected by the BLT. The BGT, LED, and DMS collected 203 (eleven species), 460 (twelve species), and 475 individuals (nine species), respectively. The species composition ratio varied for each trap. Most species accounted for less than 10% of the total catch in each trap. The predominant species in the BLT were *Ae. vexans* (31.9%), *Ochlerotatus koreicus* (28.6%), and *Cx. pipiens* (24.9%). In the BGT, *Oc. koreicus* was captured in the highest proportion (36.9%), followed by *Cx. pipiens* (20.7%), *Ae. vexans* (17.7%), and *Ae. albopictus* (10.8%). *Ae. albopictus* was present in a relatively high proportion in the BGT compared with other traps. In the LED, *Ae. vexans* accounted for more than half of the collected individuals (50.4%), followed by *Oc. koreicus* (26.3%) and *Cx. pipiens* (16.3%). In the case of the DMS, *Cx. pipiens* (86.3%) was the only species that had a more than a 10% proportion (Table 1).

The Kruskal–Wallis test revealed a significant difference in the efficiency of the traps for the total number of female mosquitoes collected (*p* = 0.005), as well as for *Cx. pipiens* (*p* < 0.001), *Ae. vexans* (*p* = 0.005), and *Ae. albopictus* (*p* = 0.002) (Table 2). Based on these results, a Mann–Whitney U test was conducted to compare the traps. The results showed that the BLT and LED captured significantly higher female mosquitoes than the BGT (*p* < 0.001 and *p* = 0.004, respectively). In particular, the BLT exhibited the highest efficacy for capturing a large number of mosquitoes. Moreover, the proportion of *Cx. pipiens* collected by the DMS was significantly higher than that collected by the BGT and LED (*p* < 0.001 and *p* = 0.001, respectively). The LED collected *Ae. Vexans* in significantly higher numbers than the DMS (*p* = 0.002), while *Ae. albopictus* was collected in significantly higher numbers by the BGT compared with the LED (*p* = 0.004) (Table 3).

The diversity indices estimated for each trap showed that the BGT had the highest value (0.777), followed by the BLT (0.751), LED (0.650), and DMS (0.251). These results indicate that the BGT is the most efficient tool for collecting a higher number of species. However, the BGT yielded an evenness index of 49.4, whereas the BLT yielded the highest evenness index of 60.1, exhibiting better species evenness results. The LED and DMS showed an evenness index of 34.3 and 12.0, respectively (Table 4).

## 4. Discussion

Mosquito surveillance is a crucial step in the prevention and control of mosquito-borne diseases, and traps serve as key surveillance tools. Several studies have been conducted to determine the efficiency and applicability of various mosquito traps [7,8,25,26,27,28,29,30,31,32,33]. However, these comparative studies have not included the DMS, which is widely used for daily surveillance in the ROK. The present study provides a comparative analysis of four mosquito traps to evaluate their effectiveness and presents the criteria for appropriate trap selection when planning mosquito monitoring projects in the ROK.

Seven genera and sixteen species, including *Anopheles* spp., were collected by the traps used in this study. The Kruskal–Wallis test revealed significant differences in the total number of female mosquitoes collected by the four traps, particularly for *Cx. pipiens*, *Ae. vexans*, and *Ae. albopictus*.

The predominant mosquito species in the present study was *Cx. pipiens* (39.7%), followed by *Ae. vexans* (27.1%), *Oc. koreicus* (21.5%), *Cx. orientalis* (4.7%), *Ae. albopictus* (1.9%), and others (5.1%). This is consistent with the findings of our previous study, conducted in a migratory bird habitat within the same region, where the predominant species was *Cx. pipiens* (30.1%), followed by *Ae. vexans* (21.1%), *Armigeres subalbatus* (18.4%), *Oc. koreicus* (9.1%), *Cx. tritaeniorhynchus* (7.6%), and others (14.0%) [17]. Differences in species composition are mainly attributed to the methodological approach used, including the habitat and period of collection. The previous study was conducted from March to November every two weeks, using two CDC light traps and one BGT near a migratory bird sanctuary.

The Mann–Whitney U test showed that the total number of mosquitoes captured by the BLT and LED was significantly higher than that collected by the BGT. A previous study has shown that the effectiveness of light traps in collecting mosquitoes increases in response to visual stimulation [37]. Our results also revealed that the BLT and LED, which are equipped with ultraviolet lamps, were more effective in attracting mosquitoes than the BGT, which does not have a light source. The species composition was also considerably different among the four traps, with the DMS capturing a significantly higher number of *Cx. pipiens* than the BGT or LED. In particular, *Cx. pipiens* accounted for more than 85% of the mosquitoes collected by the DMS, which is presumed to be primarily due to the emission of CO_2_ for a certain duration by the DMS. Mboera and Takken [38] observed that the trapping efficiency improved and the number of collected mosquitoes increased with rising CO_2_ levels up to approximately 500 mL/min. However, further studies are required to determine the appropriate amount of CO_2_ emissions required to improve the collection efficiency. Kim et al. [24] compared the efficiency of the BLT and LED in cowsheds near the demilitarized zone in the ROK and reported that the LED was more efficient in capturing six species, including *Ae. vexans*. In the present study as well, *Ae. vexans* was the most abundant species in the LED, and its proportion was significantly different than that in the DMS. However, no significant difference was observed between the BLT and LED, and the BGT was not significantly different from the other traps in collecting *Ae. vexans*. Several similar studies from Europe [27,28] have found no advantage in using the BGT to collect *Ae. vexans*. In the present study, the BGT captured the highest proportion of *Ae. albopictus*, exhibiting a significantly higher collection efficiency for this species than the LED. The collection efficiency of the BGT for *Ae. albopictus* has also been reported in the United States, Italy, Brazil, Kenya, China, and Germany [8,25,27,28,33,39,40].

The diversity index was highest for the BGT (1-D = 0.777), indicating a better collection efficiency, similar to that reported by European studies [27,28]. In contrast, no significant differences in species diversity were observed between the BGT and CDC black light traps in the United States [31]. Meanwhile, the evenness index was highest for the BLT (E = 60.1), indicating a more even collection of different species by this trap.

Based on only the performance of the traps, the use of the BLT and BGT appears to be the best choice for mosquito surveillance. However, depending on the purpose of monitoring and the target species, various factors such as the budget, labor force, and circumstance of trap installation should also be considered. For instance, the BLT (without dry ice) is the most commonly used tool for the long-term surveillance of vector mosquitoes such as *Anopheles* spp. (vector of vivax malaria) and *Cx. tritaeniorhynchus* (vector of Japanese encephalitis) in the ROK [18,19]. This preference stems from its supplied electric power and low maintenance requirements. For short-term projects, such as capturing *Ae. Albopictus* specifically, the BGT will be more effective, as it requires daily battery replacements and replenishments of dry ice. In addition, despite the relatively low efficiency of the LED and DMS in this study, we were able to consider the direction of the improvement and utilization of these traps. As already confirmed by Kim et al. [24], the LED can replace the BLT in indoor environments where host animals exist. Furthermore, the LED can be potentially used in the development of portable traps with batteries, as it consumes less power and is much smaller in size than ultraviolet lamps. The DMS, on the other hand, holds substantial value in its ability to monitor mosquitoes in real time. Although it has a collection bias, a precise prediction system can be established when used in urban areas where *Cx. pipiens* mainly occur. For instance, the Seoul metropolitan city has been operating a mosquito forecast system using the DMS since 2015 [41]. However, the introduction of this trap is limited by its high initial purchase cost, low mobility, and low efficiency. Therefore, for better performance, further studies are required to improve the efficiency of the DMS based on attractant use and the application of species identification technology using artificial intelligence.

This study had several limitations. First, our results may have been influenced by climatic factors. We rotated each trap for one day at a single location, whereas previous studies have used a Latin square design for reducing variables during experimentation [23,25,27,30]. However, seasonal climate change is gradual, and collection was avoided on days during extreme weather conditions to reduce data noise. Second, the short CO_2_ emission time of the DMS can only attract mosquitoes at nighttime, resulting in a collection bias for *Cx. pipiens*, a nocturnal species [42], in this study. Finally, this study was conducted only in a suburban area. The trap efficiency may differ depending on the environment and habitat.

Nevertheless, we believe that our results are sufficient to determine the trap efficiency and specificity. Our results indicated that the BLT and BGT are the most effective tools for monitoring mosquitoes. We also determined the merits, demerits, and limitations of the traps and provided guidance for planning new mosquito surveillance projects. Further studies are required to improve the efficiency and applicability of the LED and DMS.

## Figures and Tables

**Table 1:** Number (%) of female mosquitoes collected using the four traps.

Species		BLT	BGT	LED	DMS	Total
*Aedes albopictus*		6 (1.2)	22 (10.8)	1 (0.2)	3 (0.6)	32 (1.9)
*Ae. alboscutellatus*		1 (0.2)	1 (0.5)	1 (0.2)	0	3 (0.2)
*Ae. vexans*		163 (31.9)	36 (17.7)	232 (50.4)	16 (3.4)	447 (27.1)
*Anopheles* spp.		7 (1.4)	5 (2.5)	3 (0.7)	1 (0.2)	16 (1.0)
*Armigeres subalbatus*		3 (0.6)	8 (3.9)	4 (0.9)	1 (0.2)	16 (1.0)
*Coquillettidia ochracea*		0	0	1 (0.2)	0	1 (0.1)
*Culex bitaeniorhynchus*		1 (0.2)	1 (0.5)	1 (0.2)	0	3 (0.2)
*Cx. inatomii*		1 (0.2)	3 (1.5)	0	0	4 (0.2)
*Cx. orientalis*		34 (6.7)	7 (3.4)	12 (2.6)	25 (5.3)	78 (4.7)
*Cx. pipiens*		127 (24.9)	42 (20.7)	75 (16.3)	410 (86.3)	654 (39.7)
*Cx. rubensis*		1 (0.2)	0	0	0	1 (0.1)
*Cx. tritaeniorhynchus*		1 (0.2)	0	0	1 (0.2)	2 (0.1)
*Mansonia uniformis*		15 (2.9)	3 (1.5)	8 (1.7)	6 (1.3)	32 (1.9)
*Ochlerotatus hatorii*		4 (0.8)	0	1 (0.2)	0	5 (0.3)
*Oc. koreicus*		146 (28.6)	75 (36.9)	121 (26.3)	12 (2.5)	354 (21.5)
*Oc. nipponicus*		1 (0.2)	0	0	0	1 (0.1)
Total	Individuals	511	203	460	475	1649
	No. species	15	11	12	9	16

BLT, black light trap; BGT, BG-sentinel trap II; LED, light-emitting diode trap; DMS, digital mosquito monitoring system; No. species, the number of collected species.

**Table 2:** Results of Kruskal–Wallis test, indicating the efficiency of the traps.

	χ^2^ (df = 3, N = 52)	*p*-Value
Total number of female mosquitoes	12.89	0.005 *
*Culex pipiens*	19.11	<0.001 *
*Aedes vexans*	12.90	0.005 *
*Aedes albopictus*	14.51	0.002 *

* significant *p*-value (*p* < 0.05).

**Table 3:** Trap-versus-trap comparison using the Mann–Whitney U test, based on the significant results of the Kruskal-Wallis test.

Trap Comparison	Total Numbers of Female Mosquitoes		*Culex pipiens*		*Aedes vexans*		*Aedes albopictus*	
	Z-Adjusted Value	*p*-Value	Z-Adjusted Value	*p*-Value	Z-Adjusted Value	*p*-Value	Z–Adjusted Value	*p*-Value
BLT vs. BGT	−3.541	<0.001 *	−1.962	0.050	−2.020	0.044	−2.018	0.064
BLT vs. LED	−0.564	0.579	−1.030	0.311	−0.514	0.614	−1.531	0.311
BLT vs. DMS	−1.437	0.153	−2.413	0.014	−2.520	0.012	−0.905	0.511
BGT vs. LED	−2.771	0.004 *	−1.216	0.243	−2.351	0.019	−3.205	0.004 *
BGT vs. DMS	−1.489	0.139	−3.932	<0.001 *	−0.659	0.545	−2.723	0.014
LED vs. DMS	−0.821	0.418	−3.160	0.001 *	−2.957	0.002 *	−0.647	0.724

BLT, black light trap; BGT, BG-sentinel trap II; LED, light-emitting diode trap; DMS, digital mosquito monitoring system; * significant *p*-value determined using the Bonferroni method (*p* < 0.05/6 = 0.0083).

**Table 4:** The values of Simpson’s diversity and evenness index of the traps for the total number of female mosquitoes collected.

	BLT	BGT	LED	DMS
1-D (95% CI)	0.751 (0.750–0.751)	0.777 (0.775–0.780)	0.650 (0.647–0.653)	0.251 (0.245–0.256)
E	60.1	49.4	34.3	12.0

1-D, Simpson’s diversity index; CI, confidence interval; E, evenness; BLT, black light trap; BGT, BG-sentinel trap II; LED, light-emitting diode trap; DMS, digital mosquito monitoring system.

## Data Availability

The original contributions presented in the study are included in the article; further inquiries can be directed to the corresponding author.
